# The anxiety behind the screen: exploring the relationship between knowledge-based videos engagement and learning anxiety among postgraduate entrance exam candidates, mediated by self-efficacy

**DOI:** 10.3389/fpsyg.2025.1603034

**Published:** 2025-06-17

**Authors:** Kunjin Xu, Liangye Bao, Dan Zhang

**Affiliations:** ^1^Student Work Department, Fuzhou Polytechnic, Fuzhou, Fujian, China; ^2^Fuzhou Polytechnic, Fuzhou, Fujian, China; ^3^Communist Youth League Committee, Fuzhou Polytechnic, Fuzhou, Fujian, China

**Keywords:** knowledge-based videos engagement, learning anxiety, self-efficacy, postgraduate entrance exam (PEE) candidates, mediating role

## Abstract

**Background:**

Knowledge-based videos play a crucial role in preparing for postgraduate entrance exams (PEE). However, they may also contribute to learning anxiety.

**Objective:**

This study examines the relationship between knowledge-based videos engagement and learning anxiety among PEE candidates, focusing on the mediating role of self-efficacy. The findings will offer empirical guidance for using knowledge-based videos to alleviate learning anxiety.

**Methods:**

From September 4 to 30, 2024, a convenience sampling method was used to survey 466 candidates preparing for the 2025 national PEE in China. Data were collected through the Knowledge-based Videos Engagement Questionnaire, the Learning Anxiety Questionnaire, and the Self-efficacy Questionnaire. A mediation analysis was conducted using Hayes’ PROCESS macro with Bootstrap resampling (5,000 replications), and significance was assessed with 95% confidence intervals.

**Results:**

The mean learning anxiety of the knowledge video engagement group was significantly higher than that of the non-engagement group, whereas the mean self-efficacy of the engagement group was significantly lower. ANOVA revealed significant differences in learning anxiety across varying levels of knowledge-based videos engagement. Notably, no significant difference was observed between the “none” and “mild” engagement categories. However, “moderate” engagement emerged as a critical threshold, with “moderate,” “severe,” and “extremely severe” engagement correlating with significantly increased learning anxiety. Mediation analysis indicated that knowledge-based videos engagement did not directly predict learning anxiety. Nevertheless, it was found to significantly and positively predict learning anxiety through the mechanism of self-efficacy.

**Conclusion:**

Both moderate and higher levels of knowledge-based videos engagement are associated with a significant increase in learning anxiety among PEE candidates, with self-efficacy acting as a complete mediator in this relationship. It is recommended that PEE candidates should adopt balanced video usage strategies and enhance their self-efficacy to reduce anxiety and improve learning outcomes.

## 1 Introduction

The rapid advancement of digital technology has transformed videos and live broadcasts into essential platforms for the dissemination of knowledge, significantly altering the ways in which individuals acquire and learn (Prestridge et al., 2021). Knowledge-based videos, which are defined as those that focus on imparting knowledge and sharing insights (Chen and Wong, 2001), are distinct from entertainment videos that prioritize sensory experiences. Instead, knowledge-based videos concentrate on delivering academic content, sharing knowledge experiences, providing learning strategies, and fostering a positive learning mindset, thereby appealing to a diverse range of learners (Bai, 2022). Existing research on knowledge-based videos engagement has primarily focused on analyzing the fundamental characteristics of video content (Barthel et al., 2013), dissemination channels and strategies (Huang, 2022), inherent core competitiveness (Li and Guo, 2023), and the effects of knowledge dissemination (Zhao, 2022). However, there has been limited attention given to the emotional experiences and underlying issues associated with engagement in knowledge-based videos (He, 2022; Wang, 2020).

Postgraduate Entrance Exam (PEE) candidates, as a unique cohort preparing for the national graduate entrance examination, exhibit a significant eagerness for knowledge. The extensive range of knowledge-based videos available online serves as a crucial resource to fulfill this demand (Chen and Lin, 2024). The rising number of applicants for the PEE has been accompanied by a notable increase in learning anxiety among candidates, a phenomenon well-documented in prior research (Zhang and Ren, 2023). Consequently, numerous researchers (Li and Zhang, 2024; Lu, 2022) have conducted valuable investigations into strategies for alleviating the learning anxiety experienced by these individuals. However, in the context of the digital age, it is imperative to examine whether the use of media contributes to learning anxiety among candidates for postgraduate entrance examinations and to understand the mechanisms through which such anxiety may be triggered.

Within the field of psychological research, learning anxiety is defined as an individual’s negative cognitive, affective, and behavioral responses that are elicited by various factors throughout the learning process (Trifoni and Shahini, 2011). The relationship between learning anxiety and knowledge-based videos engagement is bidirectional: learning anxiety, as a natural reaction to academic pressures, drives individuals to seek effective coping mechanisms, including increased interaction with knowledge-based videos as supplementary learning resources (Lazarus and Folkman, 1984). Consequently, learning anxiety emerges as a significant factor influencing individuals’ engagement with such videos (Wu and Liu, 2024; Pekrun et al., 2009). Conversely, and of particular relevance to this study, knowledge-based videos, as prominent stimuli within the learning environment, directly affect learners’ emotional states by offering abundant resources and guidance, which can either mitigate or intensify learning anxiety (He, 2022; Wang, 2020).

A review of the existing literature indicates a notable deficiency in research concerning the effects of knowledge-based videos engagement on learning anxiety, with findings frequently exhibiting inconsistency. Certain studies (Lu, 2022; Flynn et al., 2023; Shreyas et al., 2023; Zhang and Kong, 2018) propose that viewing such videos may function as a relaxation strategy, mitigating learning anxiety and mental fatigue while simultaneously addressing knowledge needs, thereby potentially diminishing anxiety levels. In contrast, other research suggests that, although these videos may fulfill academic requirements, they could intensify and prolong anxiety related to learning (Kohler and Dietrich, 2021; Li et al., 2023; Li and Zhang, 2024; Luker et al., 1995). Adverse effects associated with video engagement, such as diminished visual acuity, neck discomfort, and poor sleep quality, may elevate anxiety levels rather than alleviate them (Gong, 2016). Moreover, the fragmented nature of knowledge presented in these videos can obstruct systematic learning, potentially leaving learners feeling disoriented, confused, anxious, and panicked (Li et al., 2023). These findings highlight the intricate relationship between knowledge-based videos engagement and learning anxiety, which can manifest in both positive and negative ways.

The Information Overload Theory, as proposed by Eppler and Mengis (2010), posits that when the volume of information received by an individual exceeds their cognitive processing capacity, it may lead to increased levels of stress and anxiety. This phenomenon is particularly salient for PEE candidates, who are required to acquire a comprehensive and nuanced understanding of extensive subject matter, predominantly through the consumption of knowledge-based videos (Chen and Lin, 2024). The breadth of the examination content, coupled with the substantial volume, density, and varied formats of knowledge-based videos—including supplementary comments, recommended links, and real-time viewer interactions such as danmaku—results in an information flow that significantly exceeds the brain’s processing capabilities. Consequently, this situation markedly elevates the risk of experiencing information overload.

From an information-processing perspective, cognitive overload can adversely affect each stage of the learning process. During the input stage, the influx of substantial amounts of information may lead to the oversight of critical details. In the processing stage, the difficulty in deeply processing information can hinder comprehension and internalization. At the storage stage, increased memory demands may negatively impact long-term retention. Finally, during the output stage, diminished efficiency in information retrieval can obstruct practical application. Furthermore, additional factors such as time constraints, uncertainty, and social comparisons regarding peers’ learning progress can further intensify this anxiety. Consequently, this study hypothesizes that knowledge-based videos engagement may serve as a positive predictor of learning anxiety among PEE candidates.

Rooted in Bandura (1977) foundational conceptualization of self-efficacy as “the belief in one’s capabilities to organize and execute the courses of action required to manage prospective situations,” subsequent theoretical advancements have systematically broadened its operational dimensions. Schunk (1991) framework of academic self-efficacy established critical boundary conditions, illustrating how perceived task difficulty and attributional patterns (e.g., effort versus ability attributions) dynamically moderate the formation of efficacy beliefs. Building upon this foundation, Zimmerman (2000) triadic model of self-regulated learning reconceptualized self-efficacy as a multilevel construct that integrates both domain-specific competence beliefs and metacognitive regulatory processes. This integration is particularly evident in the goal specification during the forethought phase and strategy optimization during self-reflection. Contemporary operationalizations (Bandura et al., 1999) thus recognize self-efficacy as a context-sensitive judgment that continuously interacts with environmental demands and cognitive appraisals.

As a situational factor, knowledge-based videos engagement can exert both positive and negative effects on self-efficacy (Chen and Wu, 2015; Egerton et al., 2022; Koko et al., 2020; Qi et al., 2024). On one hand, such engagement has the potential to enhance self-efficacy. Empirical evidence indicates that viewing pertinent knowledge-based videos can bolster self-efficacy among patients, thereby motivating them to adopt a more proactive approach to postoperative care (Zhao, 2017; Egerton et al., 2022). Familiarity with the information presented in these videos can further elevate self-efficacy in related domains (Gregory and Bannister-Tyrrell, 2017; Qi et al., 2024). Additionally, the capacity to manipulate the content—such as pausing and rewinding—can significantly enhance learners’ confidence (Seckman, 2017). Conversely, this engagement also poses the risk of undermining self-efficacy. According to Cognitive Load Theory (Sweller, 2010), the limitations of working memory capacity can hinder information processing. When PEE candidates engage with a multitude of videos, the rapid dissemination of information, complex visual elements, and the lack of a coherent structure and navigation can increase cognitive load. This increased cognitive load may divert attention and induce confusion and frustration, ultimately detrimentally impacting self-efficacy. In light of these considerations, this study hypothesizes that knowledge-based videos engagement may negatively predict self-efficacy among PEE candidates.

The Theory of Self-Efficacy, as proposed by Bandura (1989), asserts that self-efficacy is a critical determinant of learning anxiety. It influences an individual’s life and educational experiences by shaping cognitive patterns and emotional responses, which include the adoption of positive strategies, effective use of cognitive resources, and engagement in rational emotional regulation. A substantial body of research has demonstrated a significant negative correlation between self-efficacy and learning anxiety (Julia and Tobias, 2018). Enhancing an individual’s self-efficacy has been shown to be beneficial in mitigating learning anxiety (Watthanapas et al., 2019). Conversely, a lack of academic progress can lead to a decline in self-efficacy, which in turn may result in heightened learning anxiety (Putri and Prabawanto, 2019). Therefore, this study posits that self-efficacy serves as a negative predictor of learning anxiety among candidates in the PEE program.

However, the predictive role of self-efficacy in relation to learning anxiety is contingent upon its interactive mechanisms with preceding environmental stimuli, which significantly influences its explanatory power (Chemers et al., 2001). This dependency elucidates the rationale behind the characterization of self-efficacy as a mediating variable in numerous studies. The Social Cognitive Theory (Bandura, 1985) serves as the foundational framework for comprehending the mediating role of self-efficacy between knowledge-based videos engagement and learning anxiety.

The theory underscores the concept of triadic reciprocal determinism, which involves the interplay among environmental, cognitive, and behavioral factors. It posits that environmental stimuli, such as engagement with videos, do not directly produce emotional outcomes, such as learning anxiety. Instead, individual cognitive appraisals serve as the essential link connecting external stimuli to affective responses. In the context of preparing for the PEE, intensive knowledge-based videos engagement, as a prototypical environmental input, may indirectly affect candidates’ emotional experiences, particularly learning anxiety, by altering their belief systems concerning personal capabilities, or self-efficacy.

When candidates are subjected to an excessive amount of video content, the rapid delivery of information and the fragmented nature of knowledge structures frequently lead to cognitive overload (Chen and Lin, 2024). This environmental stressor activates negative appraisals of task difficulty, which in turn triggers threat evaluation mechanisms—where academic demands are perceived to surpass individuals’ coping capacities. Such cognitive assessments undermine self-efficacy and subsequently heighten anxiety as a stress response. Therefore, self-efficacy functions as a cognitive transducer, transforming the characteristics of the digital learning environment into anxiety-driven emotional states.

This study, informed by the preceding literature review, concentrates on PEE candidates and aims to examine the relationship between knowledge-based videos engagement, self-efficacy, and learning anxiety. Furthermore, it seeks to investigate the mediating role of self-efficacy in the relationship between knowledge-based videos engagement and learning anxiety. The primary objective is to furnish PEE candidates with empirical evidence that will empower them to make informed decisions regarding the utilization of knowledge-based videos, ultimately contributing to a reduction in their learning anxiety.

## 2 Method

### 2.1 Research design

This study employed a cross-sectional survey design. Between September 4 and September 30, 2024, a convenience sampling method was implemented to distribute an online questionnaire survey targeting potential participants for the national PEE in the cities of Nanchang, Fuzhou, Wuhan, and Hangzhou. Participants were required to meet specific inclusion criteria: they needed to express an intention to participate in the 2025 national PEE, provide informed consent, and engage in the survey voluntarily. The exclusion criteria encompassed individuals who did not intend to participate in the 2025 national PEE and those with anxiety disorders or other emotional disturbances. The recruitment process rigorously adhered to the principles of voluntary participation and informed consent. All participants confirmed their voluntary engagement through an electronic informed consent procedure prior to accessing the survey.

Data were collected anonymously utilizing the Questionnaire Star platform^1^, which is a widely employed tool in China for data collection, survey administration, and various research purposes. This platform offers a user-friendly interface and comprehensive functionalities for designing, distributing, and analyzing questionnaires. A QR code was generated to facilitate easy dissemination, linking directly to the content of the questionnaire. Our research team strategically employed online social platforms and relevant social resources to distribute the questionnaire. Participants completed the questionnaire after providing informed consent, and measures were implemented to ensure that each IP address could submit the questionnaire only once, thereby preventing potential duplications and safeguarding data integrity. The questionnaire included standardized survey instructions, mandating that all questions be answered to uphold respondents’ anonymity and confidentiality.

Based on the sample size requirements for exploratory factor analysis (EFA) as outlined in reference (Wu, 2010), a minimum target of 500 participants was established. This target was calculated as 5–10 times the number of questionnaire items (*k* = 46), resulting in a range of 230–460 participants, with additional provisions made for potential invalid responses. Ultimately, a total of 515 questionnaires were collected. To ensure data quality, dual-stage quality control procedures were implemented, which included the exclusion of responses with completion times less than the mean minus three standard deviations (i.e., less than 93 s), and the elimination of patterned responses characterized by 80% or more items receiving identical answers. These procedures resulted in 466 valid samples, yielding a valid response rate of 90.49%.

### 2.2 Demographic characteristics of participants

Among the 466 valid participants, 46 individuals (9.87%) who reported no engagement with knowledge-based videos were classified as the “non-engagement” group, while the remaining 420 participants (90.13%) with varying levels of engagement were categorized as the “engagement” group. A chi-square test was performed to assess the homogeneity of demographic variables between the engagement and non-engagement groups. The results indicated that none of the variables reached statistical significance (*p* > 0.05), suggesting the absence of systematic distributional differences in demographic characteristics between the groups. This finding supports the conclusion of balanced baseline characteristics between the two groups. Table 1 presents the basic demographic information of participants in each group.

**Table 1:** The basic information about the participants in each group.

Demographic Variables	Form	Participants overall (*n* = 466)		Engagement group (*n* = 420)		Non-engagement group (*n* = 41)		Paired chi-square test	
		Number (*n*)	Percentage (*%*)	Number (*n*)	Percentage (*%*)	Number (*n*)	Percentage (*%*)	*χ^2^*	*p*
Gender	Male	197	42.27	177	42.14	20	43.48	11.00	1.00
	Female	269	57.73	243	57.86	26	56.52		
Age	<22 years old	374	80.26	338	80.48	36	78.26	16.00	0.97
	22–30 years old	83	17.81	74	17.62	9	19.57		
	>30 years old	9	1.93	8	1.90	1	2.17		
^①^Residence	Urban	331	71.03	289	68.81	42	91.30	4.00	0.61
	Rural	135	28.97	131	31.19	4	8.70		
Type of School	Junior College	51	10.94	43	10.24	8	17.39	6.14	0.41
	General Undergraduate Institutions	242	51.93	231	55.00	11	23.91		
	^②^211 Institutions	89	19.10	82	19.52	7	15.22		
	^③^985 Institutions	84	18.03	64	15.24	20	43.48		
Major	Philosophy	10	2.15	8	1.90	2	4.35	28.00	1.00
	Economics	70	15.02	66	15.71	4	8.70		
	Legal studies	14	3.00	12	2.86	2	4.35		
	Pedagogical	48	10.30	39	9.29	9	19.57		
	Literary	36	7.73	33	7.86	3	6.52		
	History	15	3.22	13	3.10	2	4.35		
	Science	58	12.45	54	12.86	4	8.70		
	Engineering	70	15.02	66	15.71	4	8.70		
	Agronomy	13	2.79	10	2.38	3	6.52		
	Medicine	32	6.87	30	7.14	2	4.35		
	Management	67	14.38	62	14.76	5	10.87		
	Art	29	6.22	24	5.71	5	10.87		
	Military science	4	0.86	3	0.71	1	2.17		
Interdisciplinary (Yes/No)	Yes	158	33.91	135	32.14	23	50.00	3.00	0.73
	No	308	66.09	285	67.86	23	50.00		
Target Institutions	General Undergraduate Institutions	61	13.09	54	12.86	6	13.04	2.33	0.89
	211 Institutions	187	40.13	171	40.71	16	34.79		
	985 Institutions	218	46.78	195	46.43	24	52.17		
Add up the total		466	100.0	420	100.0	46	100.0	-	-

^*^p < 0.05, ^**^p < 0.01, ^***^p < 0.001, same below. ^①^In China, residential location information is categorized into two primary classifications: urban and rural. Urban areas are generally characterized by higher population density, more advanced economic structures, and well-developed infrastructure in comparison to rural regions. ^②^ The 211 Institutions constitute a significant initiative within China’s higher education framework. The objective of this program is to enhance the educational and research standards of approximately 100 universities, along with a selection of key academic disciplines, thereby elevating the status of some of China’s most distinguished universities. ^③^ The 985 Institutions represent an advanced iteration of the 211 project. Their objective is to create a distinguished cohort of first-class universities that achieve world-class standards, thereby positioning themselves as the apex of China’s higher education system.

### 2.3 Measures

#### 2.3.1 General information questionnaire

A self-designed questionnaire was utilized to gather demographic information, comprising seven items: gender, age, residence, type of school, major, interdisciplinary (Yes/No), and target institutions. Relevant studies (e.g., Bai, 2022; Huang, 2022; Li et al., 2023; Liu, 2022; Wang, 2020) have indicated that these demographic variables significantly impact PEE candidates’ knowledge-based videos engagement, self-efficacy, and learning anxiety. Consequently, in our subsequent analyses, these variables were considered as control variables.

#### 2.3.2 Knowledge-based videos engagement questionnaire

A self-constructed questionnaire (the complete questionnaire is included in the Appendix) was employed to assess the level of knowledge-based videos engagement among PEE candidates during their examination preparation. The questionnaire comprised eight items, each rated on a 5-point Likert scale, which ranged from ‘none’ to ‘extremely severe.’ Specifically, “None” was assigned a score of 1 point, indicating no engagement; “Mild” received 2 points, reflecting low engagement; “Moderate” was scored at 3 points, denoting medium engagement; “Severe” was allocated 4 points, representing high engagement; and “Extremely Severe” was given a score of 5 points, indicating excessive engagement. Participants who indicated “1 none” for the first item, signifying no engagement with the videos, were instructed to bypass the subsequent related questions. Item analysis demonstrated the significant discriminatory power of each question. The reliability of the questionnaire was confirmed with a Cronbach’s *α* of 0.79, indicating good internal consistency. Furthermore, its validity was supported by a Kaiser-Meyer-Olkin (KMO) value of 0.78, suggesting that the data were appropriate for factor analysis. Additionally, the highly significant chi-square result from Bartlett’s test of sphericity (*p* < 0.001) further validated the suitability of conducting factor analysis. All items in the factor analysis exhibited commonalities exceeding 0.5, indicating strong factor loadings.

It is essential to acknowledge that, due to the inherent individual variability in perceptual standards concerning media engagement experiences, this study operationalized knowledge-based videos engagement solely through self-reported measures. Instead of utilizing objective metrics, such as viewing duration or frequency for standardized quantification, we emphasized participants’ self-assessments of their subjective experiences and perceived intensity of engagement.

#### 2.3.3 PEE learning anxiety questionnaire

The PEE Learning Anxiety Questionnaire, developed by Shen (2006), was employed in this study. Originally comprising 16 items, the questionnaire utilized a 5-point Likert scale, with higher scores reflecting increased levels of learning anxiety. Subsequent evaluations confirmed the reliability and validity of the questionnaire. The Cronbach’s *α* coefficient, which assesses internal consistency, was determined to be 0.84, indicating a strong internal coherence of the instrument. The Kaiser-Meyer-Olkin (KMO) measure of sampling adequacy was recorded at 0.90, suggesting that the data were appropriate for factor analysis. Furthermore, Bartlett’s test of sphericity produced a chi-square value of 1266.36 (*p* < 0.001), further validating the suitability of the data for factor analysis. Collectively, these findings substantiate the questionnaire’s robust psychometric properties, thereby ensuring its reliability and validity for the research.

#### 2.3.4 Self-efficacy questionnaire

The Academic Self-Efficacy Questionnaire, as adapted by Liang (2000), synthesizes insights from multiple foundational sources, including the Academic Self-Efficacy Questionnaire developed by Pintrich and Groot (1990), the Achievement Goal Theory proposed by Ames (1992), and the Achievement Attribution Questionnaire created by Weiner (1985). The initial assessment consisted of 22 items, each evaluated using a 5-point Likert scale, with higher scores indicating greater levels of self-efficacy. The questionnaire demonstrates a Cronbach’s α coefficient of 0.86, indicating a high level of internal consistency. Furthermore, a Kaiser-Meyer-Olkin (KMO) value of 0.92 underscores the data’s strong appropriateness for factor analysis, while the significant chi-square result (*p* < 0.001) from Bartlett’s test of sphericity validates the suitability of conducting factor analysis. These robust psychometric properties substantiate the questionnaire’s reliability and validity for the study.

### 2.4 Statistical analysis

Data analysis was conducted utilizing SPSS version 23.0. A paired chi-square test was employed to assess the homogeneity of participants between the engagement and non-engagement groups. The Kolmogorov–Smirnov (K-S) test was utilized to perform normality testing on the data, while Harman’s single-factor method was applied to evaluate potential common method bias in the collected data. To examine differences in learning anxiety and self-efficacy among PEE candidates with varying levels of knowledge-based videos engagement, an analysis of variance (ANOVA) was performed, followed by *post hoc* multiple comparisons using the Least Significant Difference (LSD) method. A partial correlation analysis was conducted to investigate the relationships among knowledge-based videos engagement, self-efficacy, and learning anxiety. Furthermore, a mediation analysis was conducted employing Hayes’ PROCESS macro, which utilized the Bootstrap resampling method with 5,000 replications. Significance was evaluated through the application of 95% confidence intervals.

## 3 Results

### 3.1 Assessment of normality and common method bias

In this study, the Kolmogorov–Smirnov (K-S) test was utilized to evaluate the normality of the questionnaire data. The findings revealed that the absolute values of kurtosis for professional identity, self-control, and unethical professional behavior were 0.816, 0.587, and 1.105, respectively, while the absolute values of skewness were 0.163, 0.072, and 0.495, respectively. Drezner and Zerom (2010) contend that the criteria for normality tests are typically stringent, and absolute normality is seldom attained. According to established guidelines, if the absolute value of kurtosis is less than 10 and the absolute value of skewness is less than 3, the data can generally be regarded as conforming to a normal distribution. Consequently, the data in this study can be reasonably accepted as adhering to a normal distribution.

Common Method Bias is a phenomenon encountered in research when identical or similar methods are employed for data collection. This can lead to an artificial and misleading correlation among data points, which does not accurately reflect a genuine relationship between variables. Instead, it represents a bias arising from the limitations or consistency of the research methods (Podsakoff et al., 2003). In this study, we utilized Harman’s single-factor method to evaluate the presence of common method bias in the questionnaire data. The results indicated a χ^2^/df ratio of 3.82, exceeding the threshold of 3. Additionally, the Goodness of Fit Index (GFI), Comparative Fit Index (CFI), Normed Fit Index (NFI), and Non-Normed Fit Index (NNFI) values were 0.86, 0.84, 0.80, and 0.83, respectively, all falling below the desired level of 0.90. The Root Mean Square Error of Approximation (RMSEA) value was 0.18, surpassing the critical standard of 0.10, while the Root Mean Square Residual (RMR) was 0.09, deviating from the reference value of 0.05. Factor analysis revealed that the variance contribution rate of the first factor was 37.86%, which is below the expected critical value of 40% (Podsakoff et al., 2003). This suggests that the data from the questionnaire in this study could not be consolidated into a single factor, indicating good discriminability in the measurements. Therefore, there was no significant common method bias in the research data, and it is unlikely to have adversely affected the results of this study.

### 3.2 Descriptive statistics of the key variables

This study faced methodological challenges in evaluating the levels of qualitative variables. The lack of normative references in the design of the questionnaire precluded the use of traditional standardized comparisons for the interpretation of results. To mitigate this issue, the study employed the theoretical median reference approach, a method commonly utilized in the social sciences (Podsakoff et al., 2012), thereby establishing a conceptual evaluation framework. This approach leverages the theoretical median of the questionnaire items as a conceptual threshold to facilitate a comparative analysis of the measured means of knowledge-based videos engagement, learning anxiety, and self-efficacy. This methodologically adaptive strategy provided a theoretically robust interpretive framework in the absence of empirical anchoring data.

The findings indicated that the mean score for knowledge-based videos engagement among PEE candidates was 3.64 ± 1.25, which exceeds the theoretical median of 3 (as determined by the mean Z-test, z = 9.35, *p* < 0.001). Additionally, 83.69% of participants reported experiencing “moderate, severe, or extremely severe knowledge-based videos engagement during their exam preparation. These results suggest that PEE candidates exhibit a relatively high level of knowledge-based videos engagement. The average score for learning anxiety among PEE candidates was 3.23 ± 0.87, also surpassing the theoretical median of 3 (as indicated by the mean *Z*-test, *z* = 5.17, *p* < 0.001), which implies elevated levels of learning anxiety. Finally, the average self-efficacy score was 2.86 ± 1.11, which is slightly below the theoretical median of 3 (as shown by the mean *Z*-test, *z* = −4.38, *p* < 0.001), indicating moderate to somewhat low levels of self-efficacy among PEE candidates.

### 3.3 Comparing learning anxiety and self-efficacy among PEE candidates with varied levels of knowledge-based videos engagement

#### 3.3.1 Comparison between the “engagement” and “none-engagement” groups

A z-test was performed to compare the mean values of learning anxiety and self-efficacy between the “engagement” and “none engagement” groups, employing a 95% confidence level and a significance threshold of *p* < 0.05 to reject the null hypothesis. The results, as presented in Table 2, demonstrate that the mean learning anxiety score was significantly higher in the engagement group compared to the non-engagement group (mean *z*-test: *z* = 4.61, *p* < 0.001). Conversely, the mean self-efficacy score was significantly lower in the engagement group (mean *z*-test: *z* = −0.61, *p* < 0.05).

**Table 2:** The *z*-test for the mean difference between “engagement” and “non-engagement” groups.

Variant	Engagement group (*n* = 420)	Non-engagement group (*n* = 46)	*z*	*p*
Learning anxiety	3.31 ± 0.80	2.52 ± 1.13	4.61	0.000
Self-efficacy	2.84 ± 1.10	2.95 ± 1.14	−0.61	0.027

Given the demographic similarities between the two groups, with the sole distinction being their knowledge-based videos engagement during exam preparation (the engagement group experienced varying levels of engagement, while the non-engagement group had none), it can be inferred that the observed differences in learning anxiety and self-efficacy are primarily attributable to the variations in knowledge-based videos engagement. This finding suggests that knowledge-based videos engagement is a significant factor contributing to increased learning anxiety and decreased self-efficacy among PEE candidates.

#### 3.3.2 Comparison among different levels of engagement

The analysis of variance displayed in Table 3 reveals significant differences in learning anxiety and self-efficacy among PEE candidates with differing levels of engagement (*F* = 14.99 and *F* = 7.92, respectively, both *p* < 0.001). Subsequent *post hoc* multiple comparisons employing the Least Significant Difference (LSD) method indicated that:

**Table 3:** Differences in learning anxiety and self-efficacy at different levels of knowledge-based videos engagement.

Engagement level	Learning anxiety (M ± SD)	Self-efficacy (M ± SD)
1 None (*n* = 46)	2.52 ± 1.13	3.42 ± 1.31
2 Mild (*n* = 30)	2.71 ± 1.12	3.32 ± 1.08
3 Moderate (*n* = 113)	3.23 ± 0.73	2.88 ± 1.13
4 Severe (*n* = 134)	3.36 ± 0.75	2.89 ± 1.11
5 Extremely severe (*n* = 143)	3.45 ± 0.76	2.62 ± 1.06
*F*	14.99^***^	7.92^***^
*LSD*	3 > 1;4 > 1;5 > 1; 3 > 2;4 > 2;5 > 2;5 > 3	1 > 3; 1 > 4; 1 > 5; 2 > 3; 2 > 4; 2 > 5; 3 > 5

In the LSD *post hoc* test, report only statistically significant differences (*p* < 0.05). ^*^p < 0.05, ^**^p < 0.01, ^***^p < 0.001, same below.

**Figure 1:**
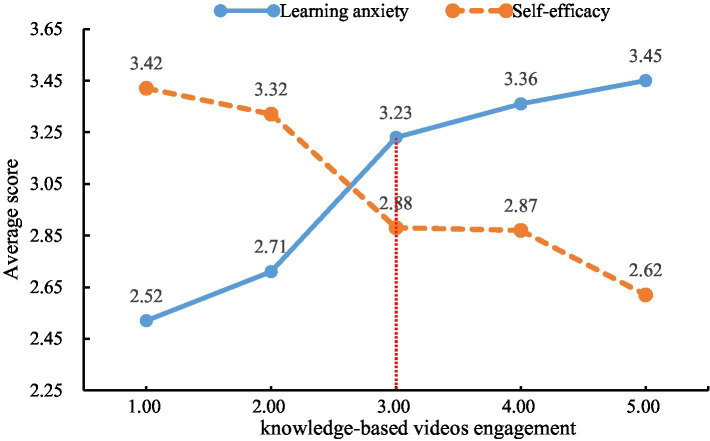
Line graphs of engagement level versus learning anxiety and self-efficacy. The dashed red line marks the critical threshold where moderate engagement with knowledge-based videos leads to significant changes in learning anxiety and self-efficacy.

In the context of learning anxiety among PEE candidates, while no statistically significant differences were observed between “none” and “mild” engagement (MD = 0.19, 95% CI [−0.05, 0.43], *p* = 0.121), a critical threshold emerged at the “moderate” level, beyond which learning anxiety increased significantly. LSD post-hoc tests revealed that moderate engagement (M = 3.23) was associated with markedly higher anxiety compared to both “none” (MD = 0.71, 95% CI [0.42, 1.00], p < 0.001) and “mild” engagement (MD = 0.52, 95% CI [0.17, 0.87], *p* = 0.004). Although “severe” engagement (MD = 0.13, 95% CI [−0.10, 0.36], *p* = 0.271) and “extremely severe” engagement (MD = 0.22, 95% CI [−0.01, 0.45], *p* = 0.062) showed an incremental rise in anxiety relative to moderate engagement, only the “extremely severe” group approached marginal significance. These findings collectively validate moderate engagement as the pivotal threshold for significant anxiety elevation, with anxiety intensifying progressively at higher engagement levels, peaking in the “extremely severe” category (see Figure 1). This pattern underscores that escalating engagement exacerbates learning anxiety, but only becomes statistically consequential once reaching moderate or higher intensity.

In relation to self-efficacy, LSD post-hoc analyses revealed that the “none” engagement and “mild” engagement exhibited the highest levels of self-efficacy, with no statistically significant difference between them (MD = 0.10, 95% CI [−0.34, 0.54], *p* = 0.650). In contrast, the “extremely severe” engagement demonstrated the lowest self-efficacy (*M* = 2.62 ± 1.06), showing significant reductions compared to both “none” engagement (MD = −0.80, 95% CI [−1.15, −0.45], *p* < 0.001) and “mild” engagement (MD = −0.70, 95% CI [−1.10, −0.30], *p* = 0.001). “Moderate” and “severe” engagement (“moderate”: MD = −0.54, 95% CI [−0.85, −0.23], *p* = 0.001; “severe”: MD = −0.53, 95% CI [−0.84, −0.22], *p* = 0.001) further highlighted a progressive decline in self-efficacy with increasing engagement intensity. The relationship between learning anxiety and self-efficacy appears to be nearly inverse; specifically, heightened engagement is associated with both elevated anxiety and a corresponding reduction in self-efficacy (see Figure 1).

### 3.4 Partial correlation analysis of the key variable

In the realm of multivariate data analysis, the correlation between two variables may be confounded or distorted by the influence of additional variables. Partial correlation analysis, as described by Podsakoff et al. (2003), addresses these confounding factors, thereby providing a more precise evaluation of the direct relationship between the variables under investigation.

As indicated in the preceding general information questionnaire, demographic variables can significantly influence key variables. Therefore, this study conducts a partial correlation analysis involving 420 PEE candidates from the engagement group. Seven demographic variables—specifically, gender, age, residence, type of school, major, interdisciplinary (Yes/No), and target institutions—are incorporated as control variables within the model. This approach aims to elucidate the intrinsic relationships among participation in knowledge-type videos, self-efficacy, and learning anxiety.

The findings indicated a positive correlation between knowledge-based videos engagement and learning anxiety (*r* = 0.19, *p* < 0.01), as well as a negative correlation between knowledge-based videos engagement and self-efficacy (*r* = −0.22, *p* < 0.01). Additionally, self-efficacy exhibited a negative correlation with learning anxiety (*r* = −0.48, *p* < 0.01). A summary of these results is presented in Table 4.

**Table 4:** Partial correlation of the key variables analysis results (*r*).

	Knowledge-based videos engagement	Learning anxiety	Self-efficacy
Knowledge-based videos engagement	1		
Learning anxiety	0.19^**^	1	
Self-efficacy	−0.22^**^	−0.48^**^	1

^*^p < 0.05, ^**^p < 0.01, ^***^p < 0.001, same below.

### 3.5 Examination of mediating effects

The study conducted an examination of the mechanisms underlying the relationship between knowledge-based videos engagement and learning anxiety among PEE candidates. It identified knowledge-based videos engagement as the independent variable, learning anxiety as the dependent variable, and self-efficacy as the mediating variable. After controlling for demographic factors as covariates, the Process program was utilized to analyze the mediating role of self-efficacy. This program, developed by Andrew F. Hayes, is a plug-in specifically designed for mediation and moderation analysis, enhancing the capabilities of traditional statistical software such as SPSS and SAS (Wen and Ye, 2014).

**Figure 2:**
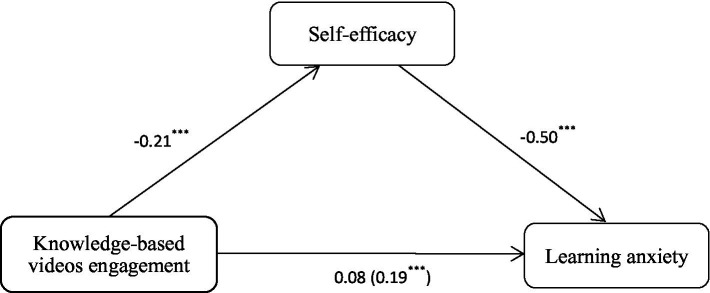
Relationships among the study constructs.

The results are presented in Table 5 and Figure 2. Model 1 demonstrated that knowledge-based videos engagement had a significant positive predictive effect on learning anxiety (*β* = 0.19, *p* < 0.001), with a 95% confidence interval of [0.11, 0.27] that did not include zero. This finding indicates that for every one standard deviation increase in engagement, learning anxiety levels increased by an average of 0.19 standard deviations, with the model accounting for 14% of the variance (*R*^2^ = 0.14). Model 2 further revealed that knowledge-based videos engagement significantly and negatively predicted self-efficacy (*β* = −0.21, *p* < 0.001; 95% CI [−0.31, −0.11]), suggesting that higher engagement was associated with lower self-efficacy. The explanatory power of the model increased to 26%. When both knowledge-based videos engagement and self-efficacy were included in Model 3, self-efficacy exhibited a strong and significant negative predictive effect on learning anxiety (*β* = −0.50, *p* < 0.001; 95% CI [−0.56, −0.44]), while the direct effect of engagement became non-significant (*β* = 0.08, *p* = 0.051; 95% CI [−0.00, 0.16]). The overall explanatory power of the model rose to 33%.

**Table 5:** Regression analysis of relationships between the key variables (*n* = 420).

	Learning anxiety(Model 1)					Self-efficacy(Model 2)					Learning anxiety(Model 3)				
	*β*	*SE*	*t*	*p*	*95%CI*	*β*	*SE*	*t*	*p*	*95%CI*	*β*	*SE*	*t*	*p*	*95%CI*
Constant	–	0.33	8.77	0.000	–	–	0.44	11.94	0.000		–	0.34	13.96	0.000	
Knowledge-based videos engagements	0.19	0.04	3.99	0.000	[0.11,0.27]	−0.21	0.05	−4.73	0.000	[−0.31,−0.11]	0.08	0.04	1.96	0.051	[−0.00,0.16]
Self-efficacy											−0.50	0.03	−10.69	0.000	[−0.56,−0.44]
*R* ^2^	0.14					0.26					0.33				
*F*	*F* (8,411) = 8.40, *p* < 0.001					*F* (8,411) = 15.93, *P* < 0.001					*F*(9,410) = 22.23, *p* < 0.001				

The stability of the full mediation effect was further validated through the Bootstrap method. As presented in Table 6, the direct effect of knowledge-based videos engagement on learning anxiety was measured at 0.08, with a 95% confidence interval of [0.00, 0.14], which includes zero, indicating a lack of statistical significance. In contrast, the mediating effect of self-efficacy between engagement and learning anxiety was found to be 0.11, with a 95% confidence interval of [0.06, 0.16], which excludes zero, thereby confirming its statistical significance.

**Table 6:** Examination of mediating effects.

	Effect size	*SE*	*t* (*z*)	*p*	*95%CI*	Effect size proportion (%)
Aggregate effect	0.19^***^	0.04	3.99	0.000	[0.08,0.24]	100.00
Direct effect	0.08	0.04	1.96	0.051	[0.00,0.14]	0
Indirect effect	0.11^***^	0.03	3.50	0.000	[0.06,0.16]	100.00

^*^p < 0.05, ^**^p < 0.01, ^***^p < 0.001, same below.

The findings indicate that, due to the non-significant direct effect and the significant mediating effect, self-efficacy serves as a complete mediator in the relationship between knowledge-based videos engagement and learning anxiety among PEE candidates, accounting for 100% of the total effect. This outcome implies that knowledge-based videos engagement indirectly intensifies learning anxiety by significantly diminishing self-efficacy, while the direct effect is entirely mediated through this pathway.

## 4 Discussion

### 4.1 PEE candidates demonstrate a relatively high level of knowledge-based videos engagement and exhibit elevated learning anxiety

This study illustrates that PEE candidates exhibit a relatively high level of knowledge-based videos engagement. This finding is consistent with the research conducted by Chen and Lin (2024) and Wang et al. (2023). The observed engagement may be attributed to the candidates’ substantial demand for learning materials, the time constraints they face during their preparation, and their awareness of the online learning resources available to them. Given the rigorous academic and research requirements associated with the PEE, candidates necessitate extensive learning resources to ensure adequate preparation. Knowledge-based videos, as a form of multimedia learning tool, provide intuitive and engaging content that enhances comprehension and mastery of the subject matter. Moreover, due to the typically limited preparation time, candidates must adopt efficient study and review strategies, rendering knowledge-based videos essential for fulfilling their educational needs, as these resources effectively convey information. As we progress further into the digital media era, the proliferation of knowledge-based videos on online platforms is increasingly becoming integrated into individuals’ daily lives and learning environments, thereby enriching the online learning experience for PEE candidates who predominantly rely on self-directed study. Consequently, PEE candidates exhibit significant knowledge-based videos engagement throughout their preparation process.

This study further elucidates that PEE candidates experience considerable learning anxiety, a finding that is consistent with the research conducted by Gao (2019) and Wang et al. (2023). These findings suggest that the substantial pressure to succeed in the PEE, stemming from candidates competing against a large cohort for limited opportunities, leads to considerable psychological stress and anxiety. Moreover, the broad scope of PEE preparation exacerbates this anxiety, compounded by a lack of clear examination requirements and insufficient information regarding their learning progress and the performance levels of their peers (Liu, 2022; Wang et al., 2023). Whether they are students or graduates, PEE candidates are also required to balance their academic obligations with their primary employment during the preparation period. Ineffective time management can lead to an increased mental burden for PEE candidates, thereby heightening their anxiety levels.

### 4.2 Both moderate and higher levels of knowledge-based videos engagement are associated with a significant increase in learning anxiety among PEE candidates

According to the study, moderate to high levels of knowledge-based videos engagement were found to increase learning anxiety among PEE candidates, which is consistent with the research findings of Li and Guo (2023) and Li and Zhang (2024). This finding supports both the Information Overload Theory (Eppler and Mengis, 2010) and the Cognitive Load Theory (Sweller, 2010). As previously noted, knowledge-based videos engagement is crucial for PEE candidates to fulfill their learning requirements (Chen and Lin, 2024). When engagement with these videos is low, candidates may lack adequate learning resources to prepare effectively for the examination, potentially resulting in heightened anxiety. Conversely, reduced engagement allows candidates to absorb information more readily, fostering a sense of control and greater confidence in their preparation, which may lead to no significant increase in anxiety levels. As knowledge-based videos engagement increases, so too does learning anxiety among PEE candidates. From the perspective of Information Overload Theory (Eppler and Mengis, 2010), moderate to high levels of engagement may signify a potential overload, wherein candidates are inundated with a considerable amount of complex information within a brief period, complicating their ability to effectively filter and assimilate it. This situation can result in information overload, which subsequently induces stress and anxiety. Furthermore, it may lead to choice anxiety, wherein candidates experience apprehension when selecting specific videos from a diverse array of learning resources, fearing they may overlook critical information or select inappropriate materials. This uncertainty further exacerbates learning anxiety. From the perspective of Cognitive Load Theory (Sweller, 2010), when PEE candidates engage with numerous knowledge-based videos—especially those that present complex content or material that surpasses their current understanding—they are required to allocate additional cognitive resources to effectively process the information. This increased cognitive demand elevates the intrinsic cognitive load. If the instructional strategies employed in the videos are inadequate, such as lacking sufficient explanations, examples, or practical exercises, learners may struggle to integrate new knowledge into their existing cognitive frameworks, thereby intensifying the cognitive load. Prolonged or excessively high cognitive load can lead to increased stress among learners, consequently heightening their learning anxiety. They may perceive the learning tasks as more complex and challenging. If their personal cognitive and coping resources are insufficient to manage this load, anxiety levels will rise significantly. Conversely, with limited knowledge-based videos engagement, candidates can more easily assimilate the information, resulting in a weak correlation between video engagement and learning anxiety.

### 4.3 Self-efficacy fully mediates the effect of knowledge-based videos engagement on learning anxiety

This study, through a comprehensive analysis of data, demonstrates that there is no statistically significant direct association between knowledge-based videos engagement of PEE candidates and their levels of learning anxiety. Furthermore, self-efficacy is shown to fully mediate this relationship. These findings not only offer empirical support for research concerning emotional mechanisms within digital learning environments but also align closely with the foundational principles of the Social Cognitive Theory (Bandura, 1985), elucidating the psychological pathways through which learning behaviors impact emotional states.

From the perspective of the Social Cognitive Theory, the findings robustly support the central proposition that inputs require cognitive mediation to exert their effects. Knowledge-based videos, as quintessential stimuli within digital learning environments, possess dual characteristics: they present intellectual challenges through complex concepts while simultaneously posing the risk of information overload. However, such environmental stimuli do not directly dictate emotional responses; rather, they must be filtered and transformed through individual cognitive systems. Specifically, when prospective educators engage with knowledge-based videos, their self-efficacy—serving as a critical cognitive mediator—governs the appraisal and interpretation of these environmental stimuli. Candidates who successfully comprehend and internalize the content of the videos enhance their self-efficacy beliefs through mastery experiences, a positive cognitive restructuring process that regulates emotional states and mitigates learning anxiety. Conversely, when individuals perceive the video content as exceeding their capabilities, negative self-efficacy evaluations exacerbate anxiety through physiological arousal and behavioral avoidance. This dynamic interaction illustrates that external factors influence emotional outcomes solely through their subjective interpretation as mediated by self-efficacy (Mclean et al., 2014).

The identified full mediation effect carries significant theoretical and practical implications. From a theoretical standpoint, the role of self-efficacy as a central nexus linking external learning behaviors and internal emotional states offers a novel perspective for understanding learning anxiety in the digital age. This perspective suggests that anxiety fundamentally reflects learners’ cognitive appraisals of their adaptability to digital environments, rather than merely the intensity of media exposure (Lau and Lee, 2020). Practically, these findings inform educational interventions that employ dual-path strategies: cognitive restructuring, such as the implementation of scaffolded learning objectives to enhance self-efficacy, and environmental optimization, for instance, the utilization of adaptive algorithms to regulate video information density. These approaches can assist learners in establishing constructive self-assessment mechanisms during human-technology interactions, effectively disrupting the detrimental cycle of information overload, eroded self-efficacy, and heightened anxiety. Ultimately, this can promote emotional well-being and enhance academic performance within digital learning ecosystems.

## 5 Research summary

### 5.1 Conclusion

This study empirically investigates the relationship between knowledge-based videos engagement of PEE candidates and their levels of learning anxiety, while also elucidating the mediating role of self-efficacy in this relationship. The findings not only enhance the understanding of Social Cognitive Theory but also offer evidence-based recommendations for PEE candidates to effectively leverage knowledge-based videos in their learning processes. The principal conclusions are as follows:

1. PEE Candidates demonstrate relatively high levels of knowledge-based videos engagement and report heightened levels of learning anxiety.
2. In the context of the relationship between knowledge-based videos engagement and learning anxiety among PEE candidates, moderate engagement has been identified as a critical threshold. Notably, both moderate and higher levels of knowledge-based videos engagement are associated with a significant increase in learning anxiety.
3. Knowledge-based videos engagement does not directly predict learning anxiety; however, it significantly and positively predict learning anxiety through the mechanism of self-efficacy. Self-efficacy serves as a full mediator in the relationship between knowledge-based videos engagement and learning anxiety.

### 5.2 Research limitations

1. Sample size and geographic limitations. The sample size of this study is relatively small compared to the overall population of PEE candidates, and the geographic concentration of participants in Eastern China may limit the generalizability of the findings. Future research should employ multi-regional stratified sampling to enhance external validity.
2. Cross-sectional design constraints on causal inference. The current cross-sectional design captures only static associations between variables and cannot elucidate the dynamic interplay among knowledge-based videos engagement, self-efficacy, and learning anxiety. Longitudinal tracking designs or time-series analyses are recommended to clarify causal-temporal relationships and dose–response relationships between variables.
3. Undifferentiated measurement of video engagement characteristics. While the study confirms the overall effect of knowledge-based videos engagement, it does not differentiate the impacts of content attributes (e.g., difficulty gradients, interactive formats, topic preferences) or usage patterns (e.g., single-session duration, fragmented frequency). Subsequent research should integrate video analytics (e.g., eye-tracking, behavioral log mining) to develop a multidimensional engagement index.
4. Potential cultural context moderation effects. The exclusive sampling from China’s educational system may introduce culture-specific biases (e.g., amplification of anxiety by the “exam-oriented culture”). Cross-cultural comparisons (e.g., collectivist vs. individualist educational environments) are needed to test the universality of the findings.
5. Simplistic assumptions in the mediation model. Although self-efficacy has been identified as a complete mediator, other variables—such as academic background, screen fatigue, motivation for video usage, perceived social support, academic resilience, self-regulation, grit and metacognitive strategies—may collectively influence the relationship between video engagement and learning anxiety. Future studies should construct structural equation modeling (SEM) integrating multiple mediators to uncover complex mediation networks.
6. Aggregation bias in learning anxiety measurement. Current scales measure global anxiety levels during exam preparation and fail to isolate anxiety specific to knowledge-based videos engagement. We recommend developing context-specific anxiety scales (e.g., distinguishing video-induced anxiety, exam anxiety, and social comparison anxiety) or adopting ecological momentary assessment (EMA) for real-time, multidimensional measurement.
7. Limitations of the Self-Constructed Questionnaire. The exclusive reliance on a five-point subjective rating scale (“None-Mild–Moderate–Severe-Extremely Severe”) without the inclusion of objective behavioral anchors (e.g., specific time thresholds or frequency criteria) may introduce cognitive bias among participants, thereby compromising the objectivity of the behavioral data collection process. Additionally, the design of the items reflects specific cultural and contextual nuances tailored to Chinese PEE candidates, raising concerns regarding the measurement validity and applicability of the questionnaire across diverse educational contexts. Furthermore, the questionnaire exhibits several limitations, particularly its failure to differentiate between various modes of participation, such as passive viewing and active interaction. These shortcomings may obscure the underlying mechanisms associated with diverse participation behaviors. Although the self-constructed questionnaire demonstrates acceptable psychometric properties, it lacks formal validation through expert panel review, which represents a methodological constraint that necessitates caution when interpreting its content validity across diverse populations. Future research should focus on establishing a standardized protocol for questionnaire development. It is recommended to incorporate item response theory (IRT) in conjunction with the expert-consensus method, augmented by cognitive load assessment technologies, such as eye-tracking or reaction time measurement, to develop a comprehensive multi-modal validity verification system.

### 5.3 Research implications

The increased prevalence of knowledge-based videos engagement, in conjunction with the widespread incidence of learning anxiety among PEE candidates, underscores the pressing necessity for targeted interventions. Our research indicates that knowledge-based videos engagement is a significant predictor of learning anxiety, particularly at moderate to high levels of engagement, with self-efficacy acting as a crucial mediating variable.

In light of the research findings presented, we propose that the core components of Social Cognitive Theory (Bandura, 1985) be integrated with contemporary advancements in Cognitive Load Theory (Sweller, 2010) for this particular population. The development of a three-dimensional intervention model encompassing “environment-cognition-behavior,” this approach aims to offer both theoretical guidance and practical strategies for mitigating educational anxiety in the digital era. The following specific recommendations are made.

First and foremost, it is crucial to balance engagement with the use of knowledge-based videos. Promoting the judicious application of knowledge-based videos is essential. While it is acknowledged that such videos offer significant advantages for learning and preparation, it is equally important to remain vigilant regarding the potential for excessive engagement to exacerbate anxiety. To mitigate this risk, it is advisable to establish a daily viewing limit (Lu, 2022) and prioritize high-quality content from reputable sources (Li et al., 2023; Li and Zhang, 2024). Furthermore, students should be encouraged to develop structured and attainable study plans, as well as to employ a variety of learning methods to accommodate diverse learning styles (Pekrun and Stephens, 2009). It is imperative to avoid an overreliance on knowledge-based videos engagement. Additionally, providing media literacy training is crucial, as it equips students with the skills necessary to critically evaluate the content they consume. Understanding algorithmic mechanisms and recognizing media biases can empower students to prevent feelings of overwhelm and mitigate any adverse effects stemming from the content to which they are exposed (Liu, 2024).

Secondly, it is imperative to implement interventions designed to enhance self-efficacy among students. Initiatives aimed at bolstering students’ self-efficacy should be developed, which can be accomplished through the organization of seminars, lectures, and personalized tutoring sessions, all with the objective of fostering students’ confidence in their academic endeavors (Sasson and Tifferet, 2025). Furthermore, the incorporation of techniques such as goal-setting, positive self-talk, and visualization may also contribute to the improvement of self-efficacy (Xie et al., 2021).

A comprehensive strategy for reconstructing the learning environment to mitigate learning anxiety should be implemented. The integration of stress management techniques is essential for alleviating anxiety. It is advisable to incorporate practices such as mindfulness meditation, yoga, and deep-breathing exercises into daily routines, as these methods have been shown to have a positive impact on anxiety reduction (Qiu, 2022). Additionally, promoting regular physical activity can significantly enhance emotional regulation (An and Peng, 2024). The establishment of peer support networks is also crucial; such networks enable students to share experiences and strategies, fostering mutual encouragement. Peer-to-peer learning can notably diminish anxiety and cultivate a sense of community among students (Wang and Wang, 2024). Furthermore, it is imperative to ensure that students have access to professional counseling services and to encourage them to seek assistance when feeling overwhelmed, as counseling can provide effective resources for managing anxiety and stress.

By employing these strategies, educational institutions can enable PEE candidates to proficiently manage their engagements with knowledge-based videos, improve their self-efficacy, and alleviate learning anxiety, thus promoting a healthier and more productive learning environment.

## Data Availability

The original contributions presented in the study are included in the article/supplementary material, further inquiries can be directed to the corresponding authors.

## Footnotes

^1^ https://www.wjx.cn

## Appendix (Self - constructed questionnaire)

### Knowledge-based videos engagement Questionnaire

Here is a questionnaire designed to assess your level of knowledge-based videos engagement. Please rate your engagement intensity based on your subjective experiences while using knowledge-based videos during your exam preparation (1–5 represents: “None,” “Mild,” “Moderate,” “Severe,” “Extremely Severe”).

1. I watch knowledge-based videos online.
2. I follow or subscribe to channels/creators of knowledge-based videos.
3. I participate in knowledge-based videos platforms by engaging in interactions, such as leaving comments or participating in Q&A sessions.
4. I actively search for and learn from knowledge-based videos in areas of interest.
5. I choose knowledge-based videos as a resource for self-directed learning.
6. I seek out relevant educational videos to assist in finding solutions to the problems I encounter.
7. I apply the content from knowledge-based videos to real-life or work scenarios.
8. I proactively recommend valuable knowledge-based video resources to others.
